# Nettleship on the Eye

**Published:** 1900-11

**Authors:** 


NETTLESHIP ON THE EYE. New Edition. Diseases of the Eye, by Edward Nettleship, F. R. C. S., Ophthalmic Surgeon at St. Thomas' Hospital, London; Surgeon to the Royal London Ophthalmic Hospital, Etc. New (6th) American from the sixth English edition, thoroughly revised by William Campbell Posey, M. D. With a Supplement on the detection of color blindness, by William Thomson, M. D., Professor of Ophthalmology in the Jefferson Medical College, Philadelphia. Just ready. In one 12mo. volume of 562 pages, with 192 illustrations. Selections from Snellen's test-types and formulæ, and five colored plates. Cloth, $2.25 net. Lea Brothers & Co., Philadelphia and New York.

"Nettleship on the Eye" is known to almost every medical student, and is regarded as standard by every member of the profession.

The 1900 American edition is carefully and skillfully revised, and no pains have been spared, it seems, to bring it up to date.

Each chapter is complete within itself, rendering it a valuable book for reference as well as a most excellent text-book.

In the revision especial attention has been given to methods of examination and therapeutic measures. The chapters on testing for color sense and acuity of vision and hearing have been carefully revised by Prof. William Thompson. Tests for admission to the public service, army, navy and merchant marine—also tests adopted by certain cities for school children—have been added by the editor, Dr. Posey. The book is compact and practical in every sense of the word, and should be in the hands of every practitioner, student and specialist.

